# Case Report: Screening and Analysis for Brucellosis in Akesai Kazak Autonomous County, China

**DOI:** 10.4269/ajtmh.22-0802

**Published:** 2023-05-01

**Authors:** Dan Zhang, Dongyue Lv, Xiaojin Zheng, Ran Duan, Shuai Qin, Xinmin Lu, Longqi Nie, Peng Zhang, Haonan Han, Qun Duan, Junrong Liang, Meng Xiao, Huaiqi Jing, Xin Wang

**Affiliations:** ^1^ State Key Laboratory of Infectious Disease Prevention and Control, National Institute for Communicable Disease Control and Prevention, Chinese Center for Disease Control and Prevention, Beijing, China;; ^2^ Akesai Kazak Autonomous County Center for Disease Control and Prevention, Jiuquan, China;; ^3^ Akesai Kazak Autonomous County Center for Animal Husbandry and Veterinary Technical Service, Jiuquan, China

## Abstract

Brucellosis is a common zoonotic disease. For this study, the residents of Akesai Kazak Autonomous County, located in the high altitude of the Altun Mountains region of Gansu Province, were selected. These people rely on traditional animal husbandry for their main income. The prevalence of brucellosis and the change of antibody titer in this high-risk population were analyzed, and information on the epidemic in animals in the county was obtained from data records. One hundred ninety-nine persons were screened and 240 serum samples were collected. Eight persons and 27 serum samples were positive based on the rose bengal plate test, and seven persons were confirmed positive by standard agglutination test; 16,000 sheep were tested, of which 130 from nine different households were serum antibody positive. The results indicate that brucellosis seroprevalence increased among sheep and high-risk populations, and the occurrence of cases corresponded to the epidemic among animals. The incidence of human brucellosis was closely related to occupation, and the cases were mainly distributed among herdsmen and butchers. Most cases were asymptomatic or mild, and the serum antibody titers showed a high initial titer but a rapid decline in young cases, whereas those in older cases were relatively low but showed a slow decline.

## INTRODUCTION

Brucellosis is an important zoonotic disease and has a wide range of clinical manifestations but usually lacks specificity, and therefore is often ignored and misdiagnosed. If not diagnosed and treated as early as possible, brucellosis can very easily become chronic and lead to serious systemic complications, and even lead to premature delivery and abortion.^1^^–^^3^ In recent years, some studies have found that most patients and animals infected with *Brucella* are asymptomatic or have mild infections.^4^^,^^5^ However, studies on the causes of asymptomatic cases and mild infections and the changes in *Brucella* antibody titer are limited. Humans are mainly infected with *Brucella* through direct or indirect contact with infected animals and via consumption of contaminated and unsterilized animal products.^6^^,^^7^ Akesai Kazak Autonomous County is a county with animal husbandry as its main economic source and is one of the regions with a high burden of human brucellosis.^8^ We conducted a serological and molecular biological investigation and study on the high-risk population of brucellosis in the pastoral area of this county in China to find clues that reflect the pathogenic characteristics and virulence changes of brucellosis.

## CASE REPORT

From January to September 2022, we selected high-risk populations who had close contact with livestock and their products for brucellosis screening. The minimum age was 5 years old, the maximum age was 78 years old, and the average age was 45.94 ± 12.502 years old. One hundred forty-eight males and 51 females were included. Eight cases (4.20%, 8/199) with twice *Brucella* serum antibody-positive results based on the rose bengal plate test (RBPT) were diagnosed as *Brucella* serum antibody-positive cases, all of whom were male, including seven herdsmen and one butcher.

Two cases (AKS2022BS195, AKS2022BS127) reported that they had been diagnosed with brucellosis in the past, and it was detected in the other six for the first time. The previously confirmed case (AKS2022BS195) and a newly found serum-positive herdsman (AKS2022BS189) felt no discomfort. Among all cases with positive serum antibodies, several symptoms were reported: 1) fatigue (62.50%, 5/8), 2) fever (37.50%, 3/8), 3) myalgia (25.00%, 2/8), 4) sweating (12.50%, 1/8), and 5) arthralgia (12.50%, 1/8). No rashes were reported (Table 1).

**Table 1:** Basic information and symptoms of cases who had positive *Brucella* serology

Case no.	Age (years)	Sex	Occupation	Fever	Sweating	Fatigue	Myalgia	Arthralgia	Rash
AKS2022BS189	36	Male	Herdsman	No	No	No	No	No	No
AKS2022BS49	48	Male	Herdsman	No	No	Yes	No	No	No
AKS2022BS56	56	Male	Herdsman	Yes	No	Yes	Yes	Yes	No
AKS2022BS67	36	Male	Herdsman	No	No	Yes	No	No	No
AKS2022BS90	70	Male	Herdsman	Yes	Yes	Yes	No	No	No
AKS2022BS96	58	Male	Herdsman	No	No	Yes	Yes	No	No
AKS2022BS195	47	Male	Butcher	No	No	No	No	No	No
AKS2022BS127	56	Male	Herdsman	Yes	No	No	No	No	No

The white blood cell (WBC) counts and C-reactive protein (CRP) of AKS2022BS195 were restored to normal at the last clinical test. Only the WBC counts of AKS2022BS67 (10.39 × 10^9^/L) were above the reference range among the newly confirmed seropositive herdsmen who had reported routine bloodwork before treatment, but the initial erythrocyte sedimentation rate (ESR) results of all herdsmen with newly confirmed seropositive results who had reported ESR before treatment were above the reference range. The first CRP results of four herdsmen were above the reference range among newly confirmed seropositive herdsmen who had reported CRP before treatment. The first hypersensitive CRP results from three herdsmen were above the reference range among newly confirmed seropositive herdsmen who had reported hypersensitive CRP before treatment. Among them, AKS2022BS67 began to take medicine after being screened positive, and the WBC counts, ESR, and CRP in the blood returned to the normal range 30 days later.

**Figure 1.**
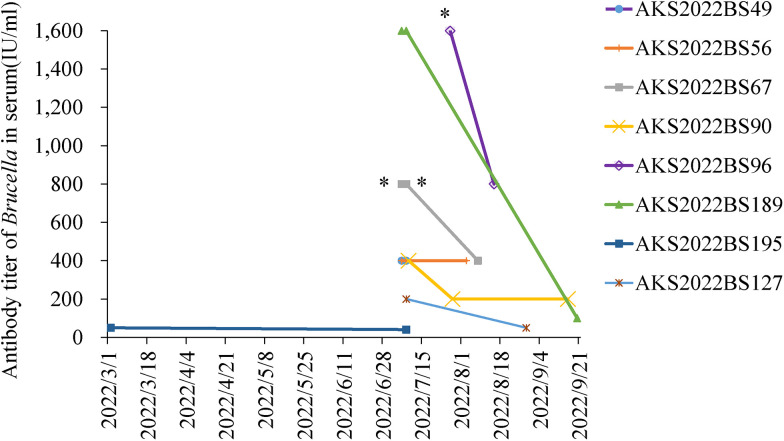
Dynamic change of serum antibody titer in *Brucella* antibody-positive cases. * = titer spots with + antibodies.

In the dynamic monitoring of *Brucella* serum antibody titer in eight people, seven cases (3.52%, 7/199) were *Brucella* serum antibody positive by standard agglutination test (without a previous history of *Brucella* infection and antibody titer ≥ 1:100 [++]; with a previous history of *Brucella* infection and antibody titer ≥ 1:50 [++]). The lowest and highest serum antibody titers of the six newly detected cases with positive *Brucella* antibody before treatment were 1:400++ and 1:1,600++, respectively. After diagnosis, they followed a treatment scheme mainly based on rifampicin plus doxycycline. With the passage of treatment time, the serum antibody titer of four cases (AKS2022BS189, AKS2022BS90, AKS2022BS67, AKS2022BS96) showed a decreasing trend. The rate of decline of AKS2022BS189, AKS2022BS67, and AKS2022BS96 was significantly faster than that of AKS2022BS90. The antibody titer of AKS2022BS56 remained stable at 1:400++ before and 28 days after treatment (Figure 1). Among the two cases who reported a history of brucellosis at the time of primary screening, the serum antibody titer of AKS2022BS127 was 1:200++ at the local hospital 52 days before screening but had dropped to below 1:50++ at the time of primary screening. AKS2022BS195 did not take any treatment within 128 days. This individual’s titer decreased from 1:50++ at the beginning of screening to 1:40++ at 129 days (Figure 1).

Nucleic acids were extracted (Blood & Tissue Kit, Qiagen 69506, Hilden, Germany) from the blood of human cases that was positive by RBPT. 16S ribosomal RNA gene polymerase chain reaction (PCR) was performed and the PCR results were all negative.

By analyzing the brucellosis test data of sheep in the county, a total of 16,000 sheep from 150 households were tested from January to September 2022, and 130 from nine different households were positive for *Brucella* serum antibody. The seroprevalence of brucellosis was 0.813% (130/16,000).

## DISCUSSION

This study shows that serum antibody-positive cases in Akesai Kazak Autonomous County are mainly distributed among herdsmen and butchers. The increase in seroprevalence of brucellosis of the local livestock corresponds to the increase in the high-risk population, so the epidemic among animals is the main reason for the increase in human cases. The eight seropositive cases found in this study were all male, which is consistent with previous studies.^7^^,^^9^ It shows that male high-risk groups have more exposure opportunities, and so male high-risk groups are the most susceptible group.

Different from the study in which asymptomatic cases developed symptoms in a short time,^10^ 66.67% (4/6) of the newly detected *Brucella* seropositive cases were mild and asymptomatic in this study. This may be related to taking the medication immediately after screening and diagnosis, and so for asymptomatic and mild cases with positive serum, in addition to strengthening monitoring and follow-up, prophylactic medication is also of great significance. Furthermore, the possibility that the virulence of the prevalent *Brucella* has decreased cannot be ruled out. The reduction in virulence is of great significance for the colonization and long-term existence of *Brucella* in the host.^11^ On the one hand, *Brucella* can escape the host’s innate immune recognition capability by changing virulence factors^12^; on the other hand, it can also lead to a reduction in immune responses by inducing cytokines.^13^^,^^14^ The RBPT used in this study could test *Brucella melitensis*, *Brucella abortus*, and *Brucella suis*. It is more meaningful to know what type of *Brucella* infects humans and animals in the area, which mainly causes asymptomatic or mild brucellosis. More attention should be paid to the pathogen in further surveillance to answer this question.

The six newly detected brucellosis seropositive cases were all acute cases. The difference among these cases was the decreased rate of serum antibody titers. The initial antibody titers of the first group were high and showed a rapid decline. The initial antibody titers of the other group were relatively low, but decreased slowly and appeared to be stationary. We believe that the slow decline of antibody titers may be due to two aspects. First, the cases in this group are older than in the other group. The elderly cases have weaker immune resistance and slower immune response. Therefore, the serum antibody titers of the elderly cases increase and decrease more slowly. Second, the possibility of acute onset of chronic brucellosis or focal disease could not be ruled out in the group with a slow decline of antibody titers.^15^

## Financial Disclosure

This work was supported by National Key Research and Development Program of China 2022YFC2602203 and National Science and Technology Major Projects 2018ZX10713-003-002 and 2018ZX10713-001-002.
